# Impact of Active Surveillance Versus Chemotherapy on Recurrence in Testicular Germ Cell Tumors: A Propensity Score–Weighted Analysis

**DOI:** 10.3390/jcm15156063

**Published:** 2026-08-04

**Authors:** Ismail Onder Yılmaz, Mehmet Zubaroğlu, Sevinç Püren Yücel, Seyda Erdogan, Mehmet Gürkan Arıkan, Nebil Akdoğan, Mutlu Değer, Volkan Izol

**Affiliations:** 1 Department of Urology, Faculty of Medicine, Cukurova University, Adana 01170, Turkey; mgarikan@cu.edu.tr (M.G.A.); nakdogan@cu.edu.tr (N.A.); mdeger@cu.edu.tr (M.D.); 2 Urology Department, Tarsus State Hospital, Mersin 33400, Turkey; mzubaroglu@cu.edu.tr; 3 Department of Biostatistics and Medical Informatics, Faculty of Medicine, Cukurova University, Adana 01170, Turkey; pyucel@cu.edu.tr; 4 Department of Medical Pathology, Faculty of Medicine, Cukurova University, Adana 01170, Turkey; seydaer@cu.edu.tr; 5 Private Clinic, Cukurova Urology Center, Adana 01170, Turkey; volkanizol@yahoo.com

**Keywords:** testicular cancer, germ cell tumor, active surveillance, chemotherapy, recurrence, inverse probability of treatment weighting (IPTW), real-world data, confounding by indication

## Abstract

**Background:** Testicular germ cell tumors (TGCTs) are the most common solid malignancies in young men. In real-world clinical practice, non-random treatment allocation may result in confounding by indication, as patients with a greater disease burden are more likely to receive chemotherapy. We evaluated the association between treatment strategy and recurrence-free survival using propensity score–based inverse probability of treatment weighting (IPTW). **Methods:** We retrospectively analyzed 114 patients who underwent radical orchiectomy for TGCT between 2015 and 2024. Patients were classified into active surveillance and chemotherapy groups. Stabilized IPTW was used to balance baseline clinicopathological characteristics. Recurrence-free survival was evaluated using Kaplan–Meier analysis and Cox proportional hazards regression. Residual post-weighting imbalance was addressed by additional covariate adjustment. **Results:** During follow-up, 29 of 114 patients (25.4%) experienced recurrence. Unadjusted Kaplan–Meier analysis demonstrated no statistically significant difference in recurrence-free survival between treatment groups (log-rank *p* = 0.150). Consistently, the unadjusted Cox proportional hazards model showed no statistically significant association between treatment strategy and recurrence-free survival (HR = 1.85, 95% CI 0.79–4.34; *p* = 0.158). After IPTW, treatment strategy remained not significantly associated with recurrence-free survival (HR = 0.96, 95% CI 0.43–2.13; *p* = 0.911). Additional adjustment for residual post-weighting imbalance yielded similar results (HR = 0.94, 95% CI 0.43–2.03; *p* = 0.876). **Conclusions:** After adjustment for measured baseline differences using IPTW, treatment strategy was not statistically significantly associated with recurrence-free survival. Given the limited number of recurrence events, wide confidence intervals, residual covariate imbalance, incomplete propensity-score overlap, and evidence of non-proportional hazards, these findings should be considered exploratory and should not be interpreted as evidence of equivalence.

## 1. Introduction

Despite accounting for only about 1% of all adult neoplasms and 5% of urological tumors, testicular cancer (TC) represents a major clinical challenge, as it is the most prevalent solid malignancy among men aged 15 to 35 years. Germ cell tumors comprise the vast majority of these cases [1,2].

Thanks to modern diagnostic modalities and cisplatin-based therapies, cure rates are exceptionally high. Consequently, contemporary management strategies focus not only on maximizing survival but also on minimizing overtreatment and long-term toxicities. The therapeutic approach following radical orchiectomy is stage-dependent. While active surveillance and adjuvant chemotherapy are appropriate management options for clinical stage I disease, systemic chemotherapy remains the standard of care in the presence of nodal involvement (N+) or distant metastasis (M+) [1,3]. Consequently, treatment allocation in real-world settings is non-random, with patients harboring a higher disease burden predominantly receiving chemotherapy [1]. In observational studies, this creates marked baseline disparities between treatment arms, thereby leading to confounding by indication and selection bias [2].

Existing literature demonstrates that recurrence rates differ significantly based on histological subtype and disease stage. For seminomatous germ cell tumors, particularly in clinical stage I disease, post-orchiectomy relapse rates are approximately 12–20%, with the vast majority of recurrences occurring within the first two years [4]. Conversely, non-seminomatous germ cell tumors carry a higher risk of recurrence; among clinical stage I patients managed with surveillance, the cumulative relapse rate approaches 30%, and can escalate to 50% in the presence of lymphovascular invasion [5]. Furthermore, clinical outcomes in metastatic germ cell tumors vary significantly according to the International Germ Cell Cancer Collaborative Group (IGCCCG) risk classification—which incorporates tumor histology, metastatic spread, and serum tumor markers—resulting in a highly heterogeneous disease course. These findings underscore that histological subtype and disease extent are critical determinants of recurrence and clinical trajectory [6,7].

The present study included patients across different disease stages within a single observational cohort. Because treatment allocation was strongly associated with baseline disease burden, particularly among patients with metastatic disease, propensity score–based inverse probability of treatment weighting (IPTW) was used to improve comparability between treatment groups with respect to measured baseline characteristics [8,9].

The objective of this study was to examine the association between post-orchiectomy management strategy and recurrence-free survival using propensity score–based IPTW, with additional adjustment for covariates showing residual post-weighting imbalance.

## 2. Materials and Methods

### 2.1. Patient Selection and Data Source

This retrospective study included patients who underwent radical orchiectomy and were pathologically diagnosed with testicular germ cell tumors between January 2015 and June 2024. The study cohort was identified using the Testicular Tumor Database of the Department of Urology at Çukurova University Faculty of Medicine.

### 2.2. Inclusion and Exclusion Criteria

Inclusion criteria comprised a history of radical orchiectomy, a histopathological diagnosis of a testicular germ cell tumor (seminoma or non-seminoma), and adequate postoperative clinical follow-up. Patients with incomplete medical records or insufficient follow-up were excluded. Accordingly, of the 130 initially evaluated patients, 16 were excluded, yielding a final cohort of 114 patients for the analysis.

### 2.3. Data Collection and Evaluated Variables

Patient data were extracted from the institutional electronic medical record system, the national Personal Health Record System (E-Nabız), and the National Death Notification System. Baseline demographic characteristics were recorded for all patients, alongside clinicopathological variables including histological subtype, tumor size, laterality, pathological T stage (pT), nodal status (N), presence of distant metastasis (M), and preoperative serum tumor marker levels (AFP, β-hCG, LDH, and testosterone).

No missing values were present for the variables included in the analyses. Accordingly, all analyses were performed using the complete dataset, and no imputation procedures were applied.

### 2.4. Treatment Group Allocation

Patients were stratified into two principal cohorts based on their primary post-orchiectomy management strategy: Active Surveillance and Chemotherapy. Treatment allocation—whether an indication for chemotherapy or surveillance—was determined through multidisciplinary tumor board consensus and shared decision-making, incorporating clinical stage, tumor marker kinetics, pathological risk factors, and contemporary urological guidelines (EAU/AUA).

### 2.5. Clinical Follow-Up and Endpoints

The primary endpoint was recurrence-free survival, defined as the time from radical orchiectomy to the first documented disease recurrence. Patients who did not experience recurrence were censored at the date of their last clinical follow-up. Recurrence was defined as the radiological detection of new metastatic lesions, including nodal or visceral lesions, on cross-sectional imaging (computed tomography or magnetic resonance imaging) and/or an unexplained persistent elevation of previously normalized postoperative serum tumor markers (AFP or β-hCG).

### 2.6. Ethical Approval

Ethical approval was granted by the Research Ethics Committee of Çukurova University Faculty of Medicine on 13 March 2026 (Meeting No. 164, Decision No. 85). This study was conducted in strict adherence to all relevant guidelines and regulations. Research methodologies complied with the ethical principles of the Declaration of Helsinki, with comprehensive measures implemented to safeguard participant health, confidentiality, and rights. Owing to the retrospective nature of the study, the requirement for informed consent was waived by the ethics committee. Accordingly, informed consent was not obtained, and no direct or indirect patient contact occurred.

### 2.7. Statistical Analysis

Normality of continuous variables was assessed using the Kolmogorov–Smirnov test. Categorical variables were summarized as counts and percentages, whereas continuous variables were summarized as medians and interquartile ranges (Q1–Q3).

To reduce potential confounding and selection bias between the chemotherapy and active surveillance groups, stabilized inverse probability of treatment weighting (IPTW) based on propensity scores was applied. Propensity scores were estimated using multivariable logistic regression based on the baseline covariates listed in Table 1. Continuous covariates were modeled as linear terms, whereas categorical variables were entered as indicator variables. No interaction terms or spline functions were included because the primary objective of the propensity score model was to achieve covariate balance rather than maximize predictive performance, and a more complex model was not considered appropriate given the modest sample size. Model discrimination was assessed using the area under the receiver operating characteristic curve (C-statistic).

Stabilized inverse probability of treatment weights (IPTW) were calculated using the marginal probability of the observed treatment assignment in the numerator to generate a weighted pseudo-population. The positivity assumption was evaluated by examining the overlap of the propensity score distributions between treatment groups, the empirical common-support region, and the distribution of the stabilized IPTW weights. Covariate balance before and after weighting was assessed using absolute standardized mean differences (|SMD|) and presented graphically. Adequate balance was defined as |SMD| < 0.10 according to the recommendations of Austin [10]. No truncation or trimming of the stabilized weights was applied in the primary analysis. The effective sample size after weighting was calculated to quantify the reduction in precision associated with the weight distribution.

Cox proportional hazards regression models were used to assess the association between treatment strategy and recurrence-free survival. For the weighted analysis, robust sandwich variance estimators were utilized with unadjusted and stabilized IPTW-weighted Cox models constructed. Further factors with absolute standardized mean difference (|SMD|) ≥ 0.10 after weighting were included in the weighted Cox model to control for remaining post-weighting imbalance. Results were expressed as hazard ratios (HR) with 95% confidence intervals (CI).

Scaled Schoenfeld residuals were used to assess the proportional hazards assumption. Sensitivity analyses were conducted to assess the effect of non-proportional hazards for the treatment variable. First, a time-dependent Cox proportional hazards model with an interaction term of treatment-by-log(time) was fitted to assess whether the treatment effect varied over time. Second, an exploratory piecewise Cox analysis was conducted using clinically defined intervals of 0–24 months and >24 months post-orchiectomy.

Statistical analyses were performed using IBM SPSS Statistics for Windows, Version 20.0 (IBM Corp., Armonk, NY, USA) and R software (version 4.5.1; R Foundation for Statistical Computing, Vienna, Austria). The R analyses were performed using the survival, survminer, WeightIt, cobalt, and survey packages. All statistical tests were two-sided, and a *p* value < 0.05 was considered statistically significant.

## 3. Results

### 3.1. Patient Characteristics and Propensity Score Diagnostics

**Figure 1:**
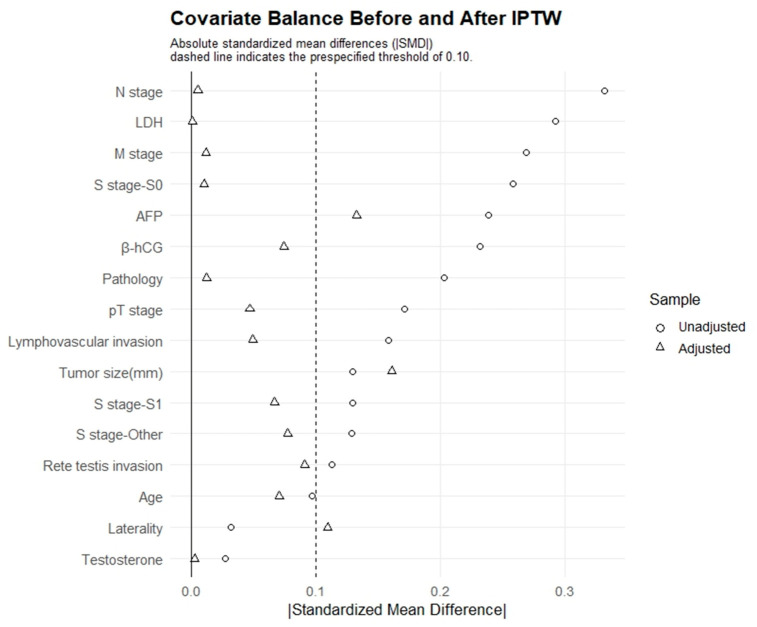
Standardized Mean Differences for Baseline Covariates Before and After IPTW.

Before weighting, the chemotherapy and active surveillance groups differed substantially in several baseline characteristics, particularly with respect to disease extent (N and M stages) and serum tumor marker profiles (e.g., LDH and AFP), demonstrating clinically relevant imbalance. After IPTW, covariate balance improved across most measured baseline characteristics, although residual imbalance remained for AFP, tumor size, and laterality (Table 1). Among the laboratory variables, β-hCG, LDH, and testosterone achieved adequate post-weighting balance, whereas residual imbalance remained for AFP, with an absolute SMD of 0.133. Most tumor-related characteristics showed improved balance after weighting; however, residual imbalance remained for tumor size and laterality, with absolute SMDs of 0.161 and 0.110, respectively. Histological subtype was also well balanced after weighting, with similar proportions of seminoma and non-seminoma between the chemotherapy and surveillance groups (seminoma: 39.4% vs. 38.1%; non-seminoma: 60.1% vs. 61.9%). The distribution of TNM staging characteristics showed no meaningful differences after weighting. The proportion of patients with pT1 was comparable between the chemotherapy and surveillance groups (61.3% vs. 56.6%). Higher pathological stages (≥pT2) were also similarly distributed (38.7% vs. 43.4%). Nodal status was also balanced, with N0 observed in 61.8% of the chemotherapy group and 62.3% of the surveillance group, and nodal involvement (N1–3) in 38.2% and 37.7%, respectively. Likewise, the frequency of distant metastasis was comparable (M0: 71.2% vs. 70.0%; M1: 28.8% vs. 30.0%). Serum tumor marker stage demonstrated similar distributions across groups (S0: 45.2% vs. 46.3%; S1: 43.6% vs. 50.3%; other: 11.2% vs. 3.4%). The distribution of standardized mean differences for baseline covariates before and after IPTW is illustrated in Figure 1. 

The vertical dashed line indicates the prespecified threshold of 0.10. Open circles (○) represent the unadjusted sample, whereas open triangles (△) represent the adjusted sample after inverse probability of treatment weighting (IPTW).

Visual inspection of the propensity-score distributions suggested substantial, although incomplete, overlap between the treatment groups across most of the propensity-score range (Supplementary Figure S1).

Distribution of estimated propensity scores in the active surveillance and chemotherapy groups are presented in density curves. Dashed vertical lines show the limits of the empirical common-support region. 

The empirical common-support interval ranged from 0.259 to 0.952, and 106 of 114 patients (93.0%) were located within this region. One patient in the active surveillance group and seven patients in the chemotherapy group were outside the empirical common-support interval. The stabilized IPTW weights had a median of 0.83 (IQR, 0.72–1.09), a minimum of [INSERT THE ACTUAL MINIMUM WEIGHT], a 95th percentile of 1.68, a 99th percentile of 2.59, and a maximum of 6.78. Only one observation had a stabilized weight greater than 5, and no observations had weights greater than 10. The effective sample size decreased from 114 before weighting to 78.5 after stabilized IPTW. The propensity-score model demonstrated moderate discrimination, with a C-statistic of 0.774 (95% CI, 0.685–0.863).

### 3.2. Follow-Up and Recurrence

The median follow-up duration from orchiectomy to the last clinical follow-up was 40.0 months (IQR, 27.3–48.8) in the active surveillance group and 39.0 months (IQR, 22.8–56.5) in the chemotherapy group, with no significant difference between the groups (*p* = 0.829). During follow-up, recurrence occurred in 29 of 114 patients (25.4%), including 7 of 37 patients (18.9%) in the active surveillance group and 22 of 77 patients (28.6%) in the chemotherapy group. Median recurrence-free survival was not reached in either treatment group.

### 3.3. Kaplan-Meier Analysis of Recurrence-Free Survival

**Figure 2:**
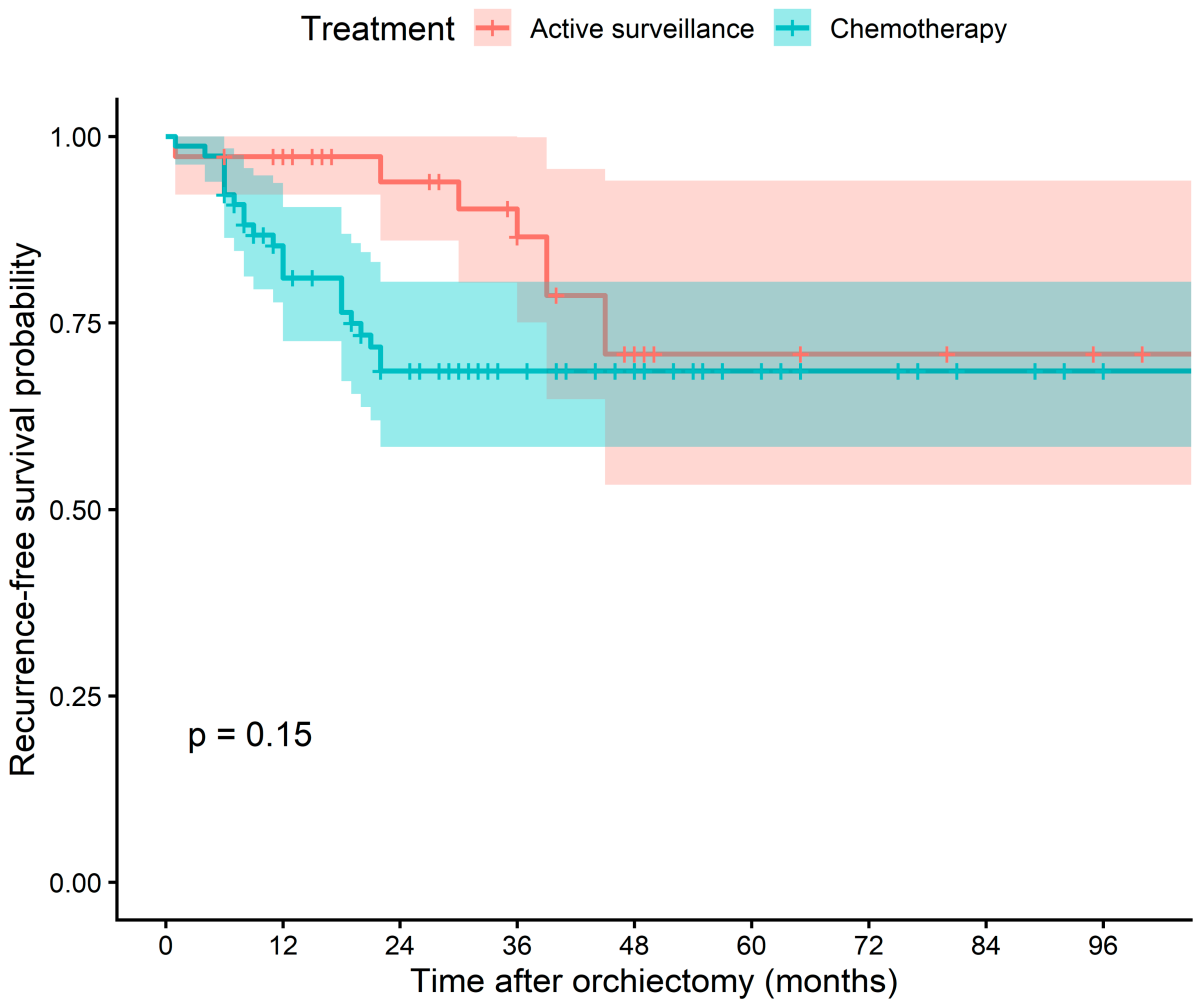
Unadjusted Kaplan–Meier estimates of recurrence-free survival according to treatment strategy.

Unweighted Kaplan–Meier analysis demonstrated numerically lower recurrence-free survival in the chemotherapy group than in the active surveillance group; however, the difference did not reach statistical significance (*p* = 0.150) (Figure 2). Estimated recurrence-free survival rates at 12, 24, and 36 months were 97.3%, 93.9%, and 86.6%, respectively, in the active surveillance group, compared with 81.0%, 68.6%, and 68.6% in the chemotherapy group.

**Figure 3:**
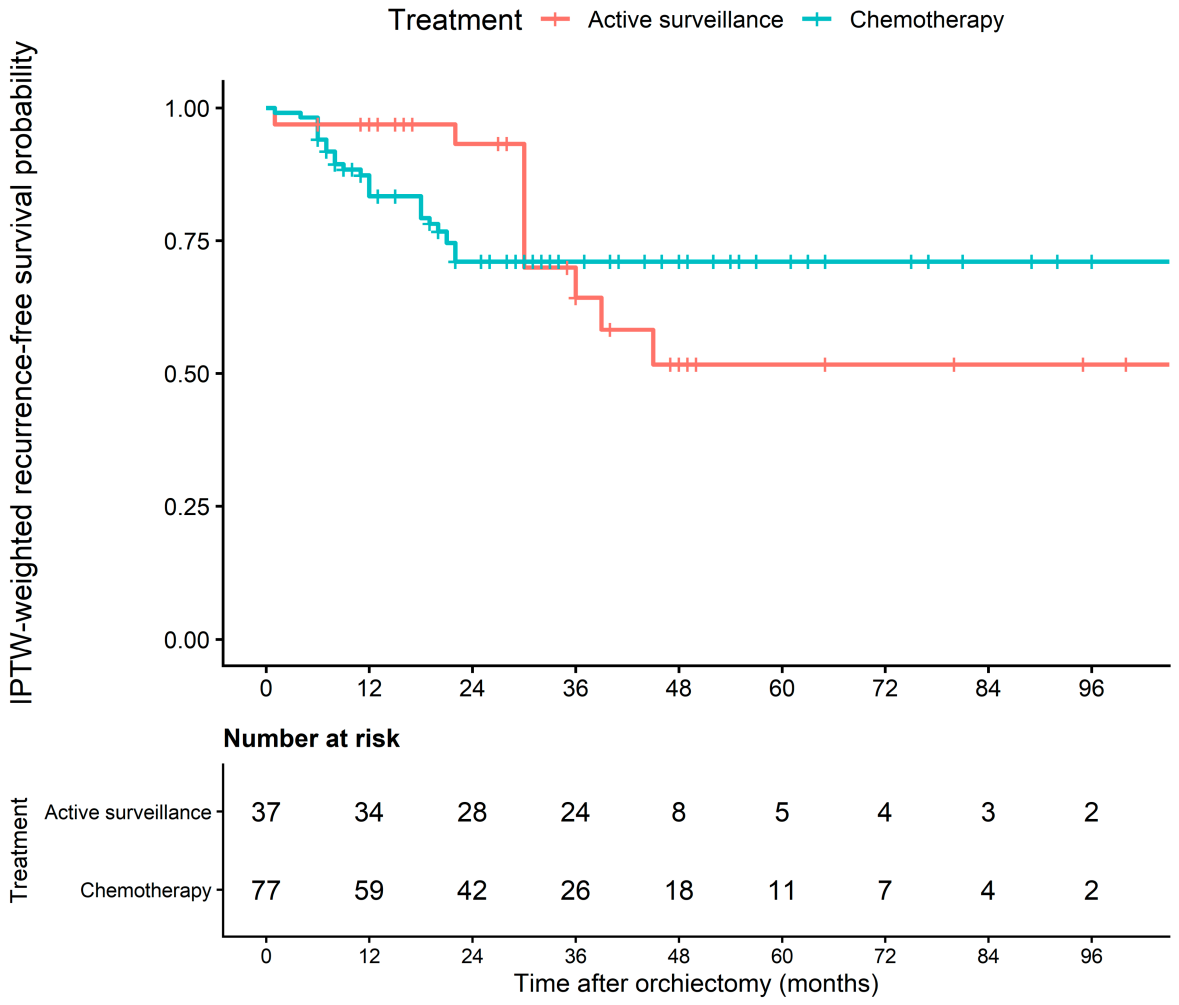
IPTW-weighted Kaplan–Meier curves for recurrence-free survival after propensity score weighting.

After propensity score weighting, weighted Kaplan–Meier curves demonstrated greater overlap between treatment groups (Figure 3). Estimated IPTW-weighted recurrence-free survival at 12, 24, and 36 months was 96.9%, 93.2%, and 64.3% in the active surveillance group and 83.3%, 71.0%, and 71.0% in the chemotherapy group.

### 3.4. Association Between Treatment Strategy and Recurrence-Free Survival

In the unadjusted Cox proportional hazards model, chemotherapy was associated with a numerically higher recurrence hazard than active surveillance; however, this association was not statistically significant (HR = 1.85, 95% CI 0.79–4.34; *p* = 0.158). After IPTW, treatment strategy was not significantly associated with recurrence-free survival (HR = 0.96, 95% CI 0.43–2.13; *p* = 0.911). Because AFP, tumor size, and laterality remained imbalanced after weighting, these variables were additionally included in the weighted Cox model. The resulting estimate was similar to that of the primary weighted model (HR = 0.94, 95% CI 0.43–2.03; *p* = 0.876), suggesting limited sensitivity of the average association estimate to residual covariate adjustment (Table 2). The residual-adjusted weighted Cox model included treatment strategy and three additional covariates. With 29 observed recurrence events and four model parameters, the observed events-per-parameter ratio was approximately 7.3, indicating that the model remained relatively data-limited.

Assessment of the proportional hazards assumption using scaled Schoenfeld residuals demonstrated evidence of non-proportional hazards for treatment (global Schoenfeld test, *p* = 0.006 in the unadjusted model and *p* = 0.001 in the IPTW-weighted model). Therefore, sensitivity analyses were performed to explore potential time-dependent treatment effects. In the time-varying Cox model, the treatment-by-log(time) interaction term was not statistically significant in any model (all *p* > 0.05), providing no clear evidence that the treatment effect changed according to a log-linear function of time. An exploratory piecewise Cox analysis demonstrated a higher recurrence hazard during the first 24 months (adjusted HR = 4.77, 95% CI 1.04–22.0; *p* = 0.045). Beyond 24 months, only five recurrence events occurred, all in the active surveillance group, precluding reliable estimation of the treatment effect because of complete separation. These exploratory analyses suggested possible temporal heterogeneity; however, the limited number of interval-specific events and the resulting imprecision precluded reliable conclusions regarding early or late treatment-associated differences.

## 4. Discussion

In the unadjusted analysis, the recurrence hazard was numerically higher in the chemotherapy group, whereas the estimated association was attenuated after IPTW adjustment for measured baseline differences. This change in the estimate may reflect the influence of measured confounding by indication in the non-randomized treatment allocation [8,9]. In clinical practice, patients with a greater disease burden are more likely to receive chemotherapy, which may contribute to an apparent difference between the treatment groups in crude analyses [1,3,11]. Nevertheless, the wide confidence intervals, residual covariate imbalance, incomplete treatment overlap, and crossing weighted Kaplan–Meier curves do not support an interpretation of equivalence between the two management strategies.

Because active surveillance is not generally considered a clinically appropriate management option for metastatic disease, the positivity assumption is less plausible in this subgroup. The full cohort was retained because the prespecified objective of the study was to evaluate the association between treatment strategy and recurrence-free survival across the entire real-world cohort. Nevertheless, retaining patients across different disease stages does not fully eliminate concerns regarding structural positivity. Therefore, the weighted estimates should be interpreted as exploratory and conditional on the observed region of common support.

Non-seminomatous histology has been associated with a higher risk of relapse in previous stage-specific studies. However, the present analysis was designed to examine the association between management strategy and recurrence-free survival and did not include a separate outcome model intended to estimate the independent prognostic contribution of histological subtype. Accordingly, no independent inference regarding histology can be drawn from the current analysis [6].

M stage contributed to the baseline differences between the treatment groups and was included in the propensity score model; however, its independent association with recurrence was not estimated in the current analysis [1,6].

The present analysis was not designed to estimate the independent prognostic contributions of lymphovascular invasion, tumor size, or rete testis invasion. These characteristics have been evaluated in previous stage-specific prognostic studies of non-seminomatous and seminomatous germ cell tumors [7,12]. Accordingly, no conclusions regarding their independent associations with recurrence can be drawn from the current treatment-focused analysis.

The application of stabilized IPTW improved balance across most measured baseline covariates in this retrospective observational cohort [8]. Given the non-randomized nature of treatment allocation in testicular tumors, treatment decisions are frequently influenced by perceived recurrence risk, thereby potentially contributing to confounding by indication [11]. Following stabilized IPTW, residual imbalance remained for AFP, tumor size, and laterality, and these variables were therefore additionally included in the weighted model. Overall, post-weighting SMDs were reduced across most baseline clinicopathological variables, although the prespecified balance threshold was not achieved for AFP, tumor size, and laterality. This analytical approach may reduce the influence of measured baseline differences; however, residual confounding from unmeasured or incompletely measured factors cannot be excluded in an observational study [8,9].

The absence of a statistically significant average association after IPTW suggests that the available data did not demonstrate a clear difference in recurrence-free survival according to treatment strategy after adjustment for measured baseline characteristics. The study provides real-world estimates of the association between post-orchiectomy management strategy and recurrence-free survival while accounting for several measured baseline differences.

Despite its methodological advantages, this study should be interpreted in the context of several limitations. First, the single-center, retrospective design may constrain the external validity and generalizability of our findings and remains susceptible to residual confounding despite the use of propensity score–based weighting. Although IPTW substantially improved balance across measured baseline characteristics, unmeasured confounding cannot be completely excluded. Second, the small number of recurrence events led to wide confidence intervals and less precise estimates of hazard ratios. Therefore, the study may have lacked the power to identify small differences in recurrence-free survival between treatment strategies. Third, the assessment of the proportional hazards assumption showed evidence of non-proportional hazards for treatment. We also performed additional sensitivity analyses including time-varying and piecewise Cox models but these should be considered as exploratory since there were only 5 recurrences >24 months and it is not possible to reliably estimate late treatment effects. Finally, our definition of recurrence encompassed both radiologically identifiable lesions and isolated, persistent elevations of serum tumor markers. While tumor markers are an integral component of surveillance in testicular germ cell tumors and may precede radiological relapse, isolated marker elevation may occasionally reflect biochemical fluctuation rather than true disease recurrence. Consequently, some degree of outcome misclassification cannot be excluded. Because recurrence subtype was not consistently documented for all patients, radiologically confirmed recurrences and isolated biochemical recurrences could not be analyzed separately, and a sensitivity analysis restricted to radiologically confirmed recurrences could not be performed.

Emerging circulating microRNAs have been investigated as potential adjunctive biomarkers for TGCT diagnosis and monitoring; however, these biomarkers were not available in the present retrospective cohort and were not included in the recurrence assessment [13].

Although IPTW improved balance across most measured covariates, residual imbalance remained for AFP, tumor size, and laterality. In addition, residual confounding related to unmeasured or incompletely recorded factors, including physician judgment, treatment-selection considerations, and patient adherence, cannot be excluded. Future prospective, multicenter, and stage-specific studies with larger cohorts, more recurrence events, and standardized recurrence classification may help clarify the association between post-orchiectomy management strategy and recurrence over time.

## 5. Conclusions

In this retrospective cohort, treatment strategy was not significantly associated with recurrence-free survival after IPTW adjustment for measured baseline differences. The attenuation of the crude association after weighting may reflect the contribution of confounding by indication and differences in measured baseline characteristics. Given the limited number of events, incomplete treatment overlap, residual covariate imbalance, and non-proportional hazards, the findings should be considered exploratory and should not be interpreted as evidence of equivalence. These observations are consistent with, but do not independently establish, support for individualized decision-making that considers disease extent, pathological characteristics, treatment-related toxicity, and patient preferences.

## Figures and Tables

**Table 1:** Comparison of Baseline Characteristics Between Groups Before and After Propensity Score Weighting.

	Original Cohort (Before IPTW)			Balanced Cohort (After IPTW)		
	Active Surveillance Group (n = 37)	Chemotherapy Group (n = 77)	$\left|SMD\right|$	Active Surveillance Group	Chemotherapy Group	$\left|SMD\right|$
Age	31.0 (27.5–41.0)	32.0 (25.0–38.0)	0.097	29.6 (23.0–40.0)	32.0 (25.0–38.2)	0.071
***Laboratory findings***						
AFP	2.6 (1.5–40.6)	16.4 (2.7–56.5)	0.238	2.6 (1.4–55.0)	9.9 (2.7–55.0)	0.133
β-hCG	4.4 (0.5–81.1)	27.0 (1.3–69.9)	0.232	2.3 (0.6–50.7)	27.0 (1.2–73.0)	0.075
LDH	240.0 (187.5–300.0)	300.0 (202.5–367.0)	0.293	255.9 (205.5–288.9)	300.0 (190.4–367.0)	0.001
Testosterone	2.6 (2.6–2.6)	2.6 (2.6–2.6)	0.028	2.6 (2.6–2.6)	2.6 (2.6–2.6)	0.003
***Tumor characteristics***						
Tumor size (mm)	38.0 (24.0–54.0)	45.0 (25.0–55.0)	0.130	30.0 (23.0–51.9)	45.0 (25.3–53.3)	0.161
Laterality, n (%) Left Right	18 (48.6) 19 (51.4)	35 (45.5) 42 (54.5)	0.032	39.2 60.1	50.2 49.8	0.110
***Histopathology***						
Pathology, n (%) Seminoma Non-seminoma	20 (54.1) 17 (45.9)	26 (33.8) 51 (66.2)	0.203	38.1 61.9	39.4 60.1	0.013
Rete testis invasion, n (%)	4 (10.8)	17 (22.1)	0.113	27.7	18.6	0.091
Lymphovascular invasion, n (%)	10 (27.0)	33 (42.9)	0.158	43.0	38.0	0.050
pT stage, n (%) pT1 ≥pT2	27 (73.0) 10 (27.0)	43 (55.8) 34 (44.2)	0.171	56.6 43.4	61.3 38.7	0.047
***Clinical staging (TNM)***						
N stage, n (%) N0 N1–3	31 (83.8) 6 (16.2)	39 (50.6) 38 (49.4)	0.331	62.3 37.7	61.8 38.2	0.005
M stage, n (%) M0 M1	33 (89.2) 4 (10.8)	48 (62.3) 29 (37.7)	0.269	70.0 30.0	71.2 28.8	0.012
S stage, n (%) S0 S1 Other	23 (62.2) 13 (35.1) 1 (2.7)	28 (36.4) 37 (48.1) 12 (15.6)	0.258 0.129 0.129	46.3 50.3 3.4	45.2 43.6 11.2	0.011 0.067 0.077

Unless otherwise specified data was expressed as median(IQR). IPTW: Inverse probability weighting, SMD: Standardized mean difference, pT stage: Pathological T stage, N stage: Nodal stage, M stage: Metastasis stage, S stage: Serum tumor marker stage.

**Table 2:** Association between treatment strategy and recurrence using Cox proportional hazards models.

Model	HR	95% CI	*p*
Unadjusted Cox	1.85	0.79–4.34	0.158
IPTW-weighted Cox	0.96	0.43–2.13	0.911
IPTW-weighted Cox + residual covariate adjustment *	0.94	0.43–2.03	0.876

* Residual adjustment included AFP, tumor size, and laterality because these variables remained minimally imbalanced after IPTW (absolute standardized mean difference ≥ 0.10).

## Data Availability

The datasets generated and/or analyzed during the current study are available from the corresponding author upon reasonable request. The data are not publicly available due to patient privacy and ethical restrictions.

## Supplementary Materials

The following supporting information can be downloaded at: https://www.mdpi.com/article/10.3390/jcm15156063/s1, Figure S1: Distribution of propensity scores by treatment group.
