# Supplementary material for: Impact of Active Surveillance Versus Chemotherapy on Recurrence in Testicular Germ Cell Tumors: A Propensity Score–Weighted Analysis
Source: J Clin Med. 2026 Aug 4;15(15):6063. doi: 10.3390/jcm15156063 (PMC13467067; doi:10.3390/jcm15156063)
Supplement: Supplementary file 1 [file jcm-15-06063-s001.zip › jcm-4419071-supplementary.pdf]

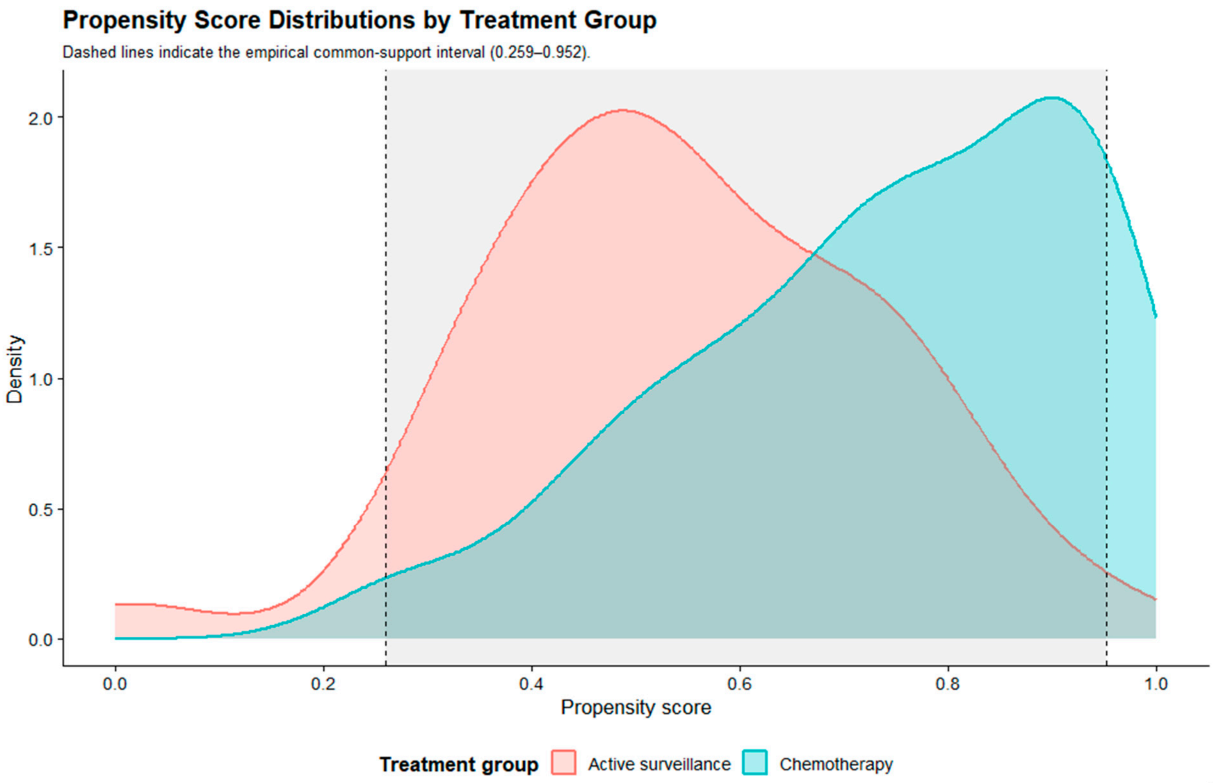

Supplementary Figure S1 Distribution of propensity scores by treatment group. Distribution of estimated propensity scores in the active surveillance and chemotherapy groups are presented in density curves. Dashed vertical lines show the limits of the empirical common-support region.
